# Real-world clinical outcomes in adult patients with Fabry disease: A 20-year retrospective observational cohort study from a single centre

**DOI:** 10.1016/j.ymgmr.2025.101229

**Published:** 2025-05-14

**Authors:** Eamon P. McCarron, Rajkumar Chinnadurai, Jonathan Meyer, Thomas Anderson, Karolina M. Stepien, Reena Sharma, Peter Woolfson, Ana Jovanovic

**Affiliations:** a Adult Inherited Metabolic Diseases, Salford Care Organisation, Northern Care Alliance NHS Foundation Trust, Stott Lane, Salford, UK; b Department of Renal Medicine, Salford Care Organisation, Northern Care Alliance NHS Foundation Trust, Salford, UK; c Division of Cardiovascular Sciences, University of Manchester, Manchester, UK; d The School of Medicine, Manchester Academic Health Sciences Centre, Manchester University, UK; e Department of Cardiology, Salford Care Organisation, Northern Care Alliance NHS Foundation Trust, Salford, UK

**Keywords:** Fabry disease, Alpha-galactosidase, Cardiometabolic, Atrial fibrillation, Mainz severity score index (MSSI)

## Abstract

**Introduction:**

Fabry disease (FD) is an X-linked lysosomal storage disorder caused by alpha-galactosidase A deficiency, leading to the accumulation of globotriaosylceramide (Gb3) and progressive damage to the cardiovascular, renal, and cerebrovascular systems.

**Aims:**

This study aimed to assess real-world clinical outcomes in FD patients, focusing on predominantly cardiovascular (CV), but also severe renal, and cerebrovascular outcomes, as well as CV and all-cause mortality. It also explored associations between age at diagnosis, Mainz Severity Score Index (MSSI), genetic mutations, and cardiometabolic risk factors such as smoking, hypertension, and obesity.

**Methods:**

A retrospective observational cohort study of 405 patients with FD was conducted by reviewing medical records from a National Centre over a 20-year period. Clinical outcomes, predominantly cardiovascular, but also severe renal and cerebrovascular events and mortality were assessed. Age at diagnosis, MSSI, and cardiometabolic risk factors were also evaluated. Statistical comparisons were performed using the Mann-Whitney *U* test and Chi-square test, with significance set at *p* < 0.05.

**Results:**

Nearly half (48 %) of patients experienced a defined clinical outcome. Higher age at diagnosis and baseline MSSI was observed in patients with poorer outcomes. The c.644 A > G (p.N215S) variant was linked with increased cardiovascular morbidity and mortality. Cardiometabolic risk factors such as smoking, hypertension, and obesity were common in patients with poorer outcomes. A high prevalence of arrhythmia, including paroxysmal atrial fibrillation (AF), was observed. Multi-morbidity was noted in deceased patients. Use of cardiometabolic therapies in at-risk groups (e.g. sodium-glucose cotransporter 2 (SGLT2) inhibitors and glucagon-like peptide-1 (GLP-1) receptor agonists) was low.

**Conclusion:**

This study highlights the clinical burden of FD, particularly among males with the c.644 A > G (p.N215S) variant. The frequent presence of cardiometabolic risk factors in patients with adverse outcomes reinforces the importance of early diagnosis, comprehensive risk evaluation, and individualised management to improve long-term prognosis.

## Highlights

• Defined outcomes occured in nearly 50% of patients
• The c.644A > G (p.N215S) variant was linked to severe disease, particularly in males.
• High AF prevalence supports the need for appropriate systematic cardiac monitoring
• Routine assessment and management of cardiometabolic risk factors should be standard care.
• Further research is required to evaluate the role of emerging cardiometabolic therapies in FD

## 1 Introduction

Fabry disease (FD, OMIM #301500) is an X-linked lysosomal storage disorder caused by pathogenic variants in the *GLA* gene, leading to a deficiency of the enzyme alpha-galactosidase A [1,2]. This enzyme deficiency leads to the accumulation of globotriaosylceramide (Gb3), resulting in progressive damage across multiple organ systems, including the cardiovascular (CV), renal, and cerebrovascular systems [3,4]. Although clinical manifestations can occur early in life, many patients, particularly males, present later with significant morbidity [[5], [6], [7]]. CV events are the leading cause of mortality in patients [2,8], closely followed by renal failure and stroke [2,[9], [10], [11]]. Common CV events include arrhythmias (e.g. ventricular tachycardia (VT)), and heart failure [12,13]. Additionally, structural heart disease and conduction abnormalities are implicated in sudden cardiac death among affected patients [14,15]. Renal involvement, which begins with proteinuria, can progress to chronic kidney disease (CKD) and end-stage kidney disease (ESKD) if untreated [10,11,16]. Cerebrovascular complications, including transient ischaemic attacks (TIA) and cerebrovascular accidents (CVA), are common, often resulting from small vessel disease secondary to Gb3 accumulation [16].

The *GLA* variants c.644A > G (p.N215S) and c.902G > A (p.R301Q) are among the most common mutations associated with later-onset disease, typically presenting with cardiac-predominant phenotypes and retaining partial alpha-galactosidase A activity, which contributes to a milder clinical course compared to classical variants that lead to early multi-organ involvement [[17], [18], [19]]. Analysis from the United Kingdom (UK) Biobank revealed that individuals carrying these mutations, particularly p.N215S, exhibited increased CV morbidity and that the prevalence of *GLA* variants in the general population may be higher than previously reported [20].However, reliable blood-based biomarkers to monitor disease progression and therapeutic response remain lacking. Residual enzyme activity often poorly correlates with disease severity, especially in female carriers, and can be influenced by therapy or mutation type [21,22]. Lysosomal Gb3 (lysoGb3) concentrations reflect systemic disease burden but are less sensitive to early-stage or organ-specific damage, vary by gender and mutation, and do not always normalise with treatment [23,24]. Various clinical scoring systems, such as the Mainz Severity Score Index (MSSI), have been developed to assess disease severity and monitor the clinical course in response to enzyme replacement therapy (ERT) [25]. The MSSI evaluates general, neurological, CV, and renal manifestations; however, its utility can be confounded by the progressive nature of the disease with ageing, which complicates comparisons across different age groups.

Given the complexity and variability of clinical manifestations, early detection and intervention are crucial. Previous research has emphasized the importance of early ERT or chaperone therapy in mitigating disease progression and improving outcomes [3,20,[26], [27], [28], [29]]. Cardiometabolic risk factors, including smoking, hypertension, obesity, are major contributors to CV and renal disease in general and can exacerbate complications in FD [30]. These factors, along with lysosomal dysfunction, may accelerate cardiac and renal complications [31]. Emerging therapies, such as sodium-glucose cotransporter 2 (SGLT2) inhibitors and glucagon-like peptide-1 (GLP-1) receptor agonists, offer cardioprotective and renoprotective benefits in broader populations; however, their efficacy in FD remains poorly studied. Therefore, further investigation into the use of these therapies in this population is essential to better understand their potential in improving disease progression and clinical outcomes.

### 1.1 Aim

To evaluate the real-world clinical outcomes of patients with FD at a National Centre, focusing on predominantly CV, but also severe renal, cerebrovascular outcomes, and CV and all-cause mortality. This study will investigate the associations between age at diagnosis, baseline MSSI, common genetic mutations, and cardiometabolic risk factors, including smoking, hypertension, and obesity, with clinical outcomes. Additionally, we will assess the use of cardiometabolic therapies (e.g. GLP-1 receptor agonists) in at-risk groups (e.g. heart failure and obesity).

## 2 Methods

### 2.1 Study design

A retrospective observational cohort study of real-world clinical outcomes was conducted by reviewing the medical records of 405 patients diagnosed with FD who attended a National Centre, with data collected from approximately 2000 to 2022 (over two decades). We systematically assessed the prevalence of clinical outcomes, predominantly CV, but also severe renal and cerebrovascular events, as well as CV and all-cause mortality. This was a within-cohort observational analysis, and no external control group was included; comparisons were made between patients with and without defined clinical outcomes. The MSSI was calculated retrospectively based on clinical features documented at or near the time of diagnosis, including general, neurological, CV, and renal domains, in accordance with established scoring criteria [25].

The inclusion criteria encompassed adults aged 18 years or older with a confirmed genetic diagnosis of FD and patients with complete records within the specified timeframe. Those with incomplete data were excluded. Descriptive analyses were performed to identify associations between baseline MSSI, age at diagnosis, genetic mutations, and cardiometabolic risk factors such as smoking status, hypertension, and body mass index (BMI) with the specified clinical outcomes. The study adhered to ethical guidelines, ensuring patient confidentiality and data protection throughout the analysis. Institutional approval (study reference: S19MET08-S) and Health Research Authority approval (identification number: 262706, reference: 19/HRA/5221) were obtained, and the study was exempt from review by a regional ethics committee.

### 2.2 Diagnosis of Faby disease

Patients were referred to the National Centre through several pathways: following a confirmed diagnosis of FD made at local or regional centres, based on clinical suspicion requiring further evaluation, or as part of cascade screening in families with known pathogenic *GLA* variants. Diagnosis was typically based on reduced alpha-galactosidase A activity or elevated plasma or urinary lysoGb3, and confirmed by *GLA* gene mutational analysis. Due to the retrospective nature of the study and variability in historical medical records, it was not possible to reliably determine the timing of diagnosis relative to referral for each individual.

In most cases, diagnosis followed a stepwise approach: initial biochemical testing (reduced alpha-galactosidase A activity and/or elevated plasma or urinary lysoGb3) was followed by confirmatory *GLA* gene mutational analysis, particularly in males. In females and cases with borderline enzyme activity, molecular testing was typically performed early in the diagnostic process. While timing varied slightly across the 20-year study period, this sequential approach was generally consistent with UK clinical practice guidelines throughout.

Age at diagnosis was defined as the age at which FD was formally diagnosed based on biochemical and genetic confirmation. While age at onset of symptoms or age at first clinical outcome may differ, only age at confirmed diagnosis was used in this analysis due to variability in the availability and reliability of historical data across the cohort.

### 2.3 Definition of clinical outcomes

Clinical outcomes were categorised into four primary areas: CV, renal, cerebrovascular, and mortality. For CV outcomes, we identified non-fatal cardiovascular events (NFCVE), including arrhythmias, atrial fibrillation (AF), non-sustained VT (NS-VT), sustained VT (S-VT), myocardial infarction (MI), and heart failure. Additionally, we documented the placement of cardiac devices, including implantable cardiac defibrillators (ICDs) and pacemakers (PPMs). Renal outcomes in this cohort have been previously described in detail, including the progression and predictors of FD nephropathy [11]. In the present study, we analysed severe outcomes, tracking the initiation of renal replacement therapies, including haemodialysis (HD), peritoneal dialysis (PD) and renal transplants (RT). Cerebrovascular outcomes included TIA and CVA. Mortality outcomes encompassed both CV and all-cause.

AF and VT were diagnosed through the analysis of heart rhythms on a cardiac monitor, electrocardiogram (ECG), or 24-h ECG. Paroxysmal AF was defined as intermittent, irregular heart rhythms with no clear P waves, resolving spontaneously within 7 days. Persistent AF appears as continuous irregular rhythms for more than 7 days, while permanent AF was consistently irregular without any periods of normal rhythm, even after attempts to revert it. Sustained VT was defined as an irregular rhythm lasting thirty seconds or shorter if the patient had symptoms. In contrast, non-sustained VT was defined as less than thirty seconds, often spontaneously reverting to a normal rhythm.

### 2.4 Cardiometabolic risk factors

Cardiometabolic risk factors (smoking history, hypertension, and BMI) were recorded categorically as present or absent at baseline, with individual baseline values also recorded to facilitate median calculation. Smoking was defined as current or recent ex-smoker at the time of diagnosis. Longitudinal data on smoking duration, cessation, or cumulative exposure were not consistently available. Hypertension was defined as a reading of ≥140/90 mmHg on more than one occasion requiring treatment. Obesity was defined as a BMI ≥30 Kg/m^2^. Diabetes was recorded as present if on appropriate medication. Hypercholesterolaemia was defined as a total cholesterol >5.5 mmol/L.

### 2.5 Statistical analysis

Statistical analyses were conducted using SPSS software (version 25), and graphical representations were produced using Microsoft Excel®. Continuous variables were summarised using medians and interquartile ranges (IQR), while categorical data were presented as frequencies and percentages. The Chi-square test was used to analyse categorical variables, and the Mann-Whitney *U* test was applied for comparisons of continuous data. Statistical significance was set at a *p*-value of <0.05. The output of the Mann-Whitney *U* test included the *U* statistic, the *Z* value, and the corresponding p-value, enabling the determination of statistically significant differences between the groups.

## 3 Results

### 3.1 Demographics

All demographic and baseline clinical data are summarised in Table 1. Most patients were diagnosed between the ages of 20 and 39 years, corresponding to the third and fourth decades of life (median MSSI 6.0 (1–15) and 14 (6.5–21) for each respective decade), with a higher proportion of females diagnosed before the age of 39. A small number of patients initially treated with chaperone therapy were later switched to ERT due to suboptimal clinical response or declining renal function. All patients receiving migalastat met renal function eligibility criteria at the time of treatment initiation. Treatment decisions throughout the cohort were made in accordance with national UK clinical guidelines at the time, taking into account individual factors such as renal function, mutation amenability, and treatment response.

**Table 1:** Demographic and baseline clinical characteristics, stratified by sex (BMI = body mass index, BP = blood pressure, ERT = enzyme replacement therapy, MSSI = Mainz Severity Score Index, CV = cardiovascular, IHD = ischaemic heart disease, TIA = transient ischaemic attack, CVA = cerebrovascular accident, AF = atrial fibrillation, eGFR = estimated glomerular filtration rate, uACR = urine albumin creatinine ratio, CKD=Chronic kidney disease, stage 3 = eGFR <59 ml/min/1.73m^2^ [32]).

	Male 182 (44.9 %)	Female 223 (55.1 %)	Total 405	p-value
**Age at diagnosis, years**	44.0 (33–54.8)	39.0 (27–54.5)	42.0 (31–55)	0.066
**BMI, Kg/m**^**2**^	25.7 (22.8–29)	26.0 (23.3–30)	25.9 (22.9–29.8)	0.214
**Obesity**	40 (22 %)	49 (22 %)	89 (22 %)	0.999
**Systolic BP, mm of Hg**	129.5 (117–143.5)	123.5 (115–136)	126.5 (116–140)	**0.006**
**Diastolic BP, mm of Hg**	77 (70–84)	76.0 (70–84)	76 (70–84)	0.897
**Hypertension**	42 (23.1 %)	39 (17.4 %)	81 (20 %)	0.162
**Diabetes mellitus**	8 (4.5 %)	4 (1.8 %)	12 (3 %)	0.125
**Hypercholesterolemia**	17 (9.3 %)	17 (7.7 %)	34 (8.4 %)	**0.535**
**Smoking history**	37 (20.3 %)	36 (16.1 %)	73 (18 %)	0.276
**ERT**	128 (70 %)	70 (31.4 %)	198 (48.9 %)	**<0.0001**
**Chaperone therapy**	55 (30 %)	32 (14.3 %)	87 (21.5 %)	**<0.0001**
**Baseline MSSI score**	18.0 (12−23)	4.0 (1–15)	12.0 (3−20)	**<0.0001**
**c.644A >** **G (p.N215S)**	70 (38.5 %)	63 (28.3 %)	133 (32.8 %)	**0.030**
**c.902G** **>** **A (p.R301Q)**	14 (7.1 %)	13 (5.8 %)	27 (6.7 %)	0.592
**CV morbidity (IHD, TIA, CVA)**	26 (14.3 %)	21 (9.4 %)	47 (11.6 %)	0.128
**Arrhythmia (AF)**	16 (8.8 %)	10 (4.5 %)	26 (6.4 %)	0.078
**eGFR, ml/**min**/1.73m**^**2**^	100 (82–113)	109 (92–120)	104 (87–118)	**0.001**
**uACR, mg/mmol**	4.7 (1.28–39.9)	1.86 (0.84–10.8)	2.6 (0.97–22.2)	**0.004**
**CKD (≥ stage 3)**	29 (15.9 %)	6 (2.7 %)	35 (8.6 %)	**<0.0001**
**All-cause mortality**	28 (15.4 %)	7 (3.1 %)	35 (8.6 %)	**0.0002**
**Follow-up**	6.9 (4.2–12.6)	6.2 (3.6–11.8)	6.4 (3.6–12.2)	0.316

### 3.2 Clinical outcomes

**Fig. 1:**
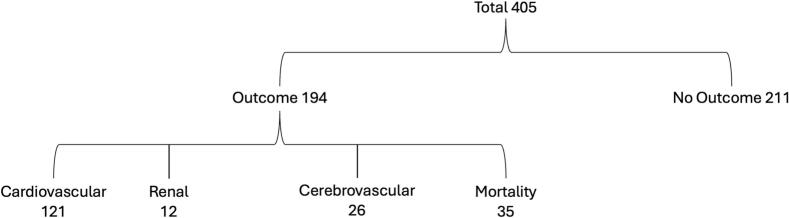
Flow diagram of patients included in the analysis.

Patients were grouped based on clinical outcomes are shown in Fig. 1. Defined clinical outcomes were recorded for each patient and are displayed in Table 2. An estimated 47.9 % (194) of patients experienced a CV, renal, cerebrovascular outcome or mortality. 52.1 % (211) of patients experienced no defined outcome. Within this group; 83.4 % (176) were female and 16.6 % (35) were male. 40.8 % (86) had the c.644 A > G (p.N215S) mutation, 6.2 % (13) c.902G > A (p.R301Q) and 6.2 % (13) c.695 T > C (p.I232T). 15.6 % (33) had hypertension, 18.0 % (38) were current/recently ex-smokers, and 3.3 % (7) had both risk factors. The median BMI was 25.3 kg/m^2^ (22.7–28.9), 13.3 % (28) had a BMI >30.0 kg/m^2^. The median MSSI was 6 (1–15), with 27.9 % (59) patients with no recorded outcome having an MSSI score of 0. Differences between the outcome and no outcome group, along with the corresponding p- values, are shown in Table 3*.*

**Table 2:** Patient outcomes by gender for cardiovascular, renal, cerebrovascular, and mortality events. (NFCVE = non-fatal cardiovascular event, NS-VT = non-sustained ventricular tachycardia, S-VT = sustained ventricular tachycardia, MI = myocardial infarction, NYHA = New York heart association, ICD = internal cardiac defibrillator, PPM = permanent pacemaker, HD = haemodialysis, PD = peritoneal dialysis, RT = renal transplant).

Outcome	Male	Female	Total
**CV**			
**Number of patients (N)**	79 (65.3 %)	42 (34.7 %)	121 (29.9 %)
**Events**	155	110	265
**NFCVE**	142	108	250
**Arrythmia**	94	44	138
**AF**	38	16	54
**NS-VT**	50	25	75
**S-VT**	6	3	9
**MI**	7	5	12
**Heart failure** **(NYHA III/IV)**	4	4	8
**Cardiac device**	37	14	51
**ICD**	18	2	20
**PPM**	19	12	31
**CV mortality**	13	2	15
**Renal**			
**N**	10 (83.3 %)	2 (16.7 %)	12 (3 %)
**Events**	14	2	16
**HD/PD**	10	2	12
**RT**	4	0	4
**Cerebrovascular**			
**N**	16 (61.5 %)	10 (38.5 %)	26 (6.4 %)
**Events**	16	10	26
**CVA**	14	5	19
**TIA**	2	5	7
**Mortality**			
**N**	28 (80 %)	7(20 %)	35(8.6 %)
**Non-CV mortality**	15	5	20

**Table 3:** Differences in baseline characteristics stratified by presence or absence of defined clinical outcome**.** (BMI = body mass index, BP = blood pressure, ERT = enzyme replacement therapy, MSSI = Mainz Severity Score Index).

	Outcome 194 (47.9 %)	No outcome 211 (52.1 %)	p-value
**Male**	133 (68.6 %)	35 (16.6 %)	**<0.0001**
**Age at diagnosis, years**	54 (43–60)	36 (23–48)	**<0.0001**
**BMI, Kg/m**^**2**^	26.7 (23–30.9)	25.4 (22.7–28.9)	**0.016**
**Obesity**	61 (31.4 %)	28 (13.3 %)	**<0.0001**
**Systolic BP, mm of Hg**	131 (119–146.5)	123 (113.5–134)	**<0.0001**
**Diastolic BP, mm of Hg**	77 (70.5–86)	76 (69–83)	0.076
**Hypertension**	48 (24.7 %)	33 (15.6 %)	**0.022**
**Smoking history**	35 (18 %)	38 (18 %)	0.993
**ERT**	102 (52.6 %)	96 (45.5 %)	0.154
**Chaperone therapy**	37 (19.1 %)	50 (23.7 %)	0.258
**Baseline MSSI score**	21 (14–27)	6 (1–15)	**<0.0001**
**c.644A >** **G (p.N215S)**	56 (28.9 %)	77 (36.5 %)	0.103
**c.902G** **>** **A (p.R301Q)**	14 (7.2 %)	13 (6.2 %)	0.670
**Follow-up (years)**	6.3 (3.7–6.4)	5.5 (3.3–11.1)	0.119

### 3.3 Age at diagnosis

**Fig. 2:**
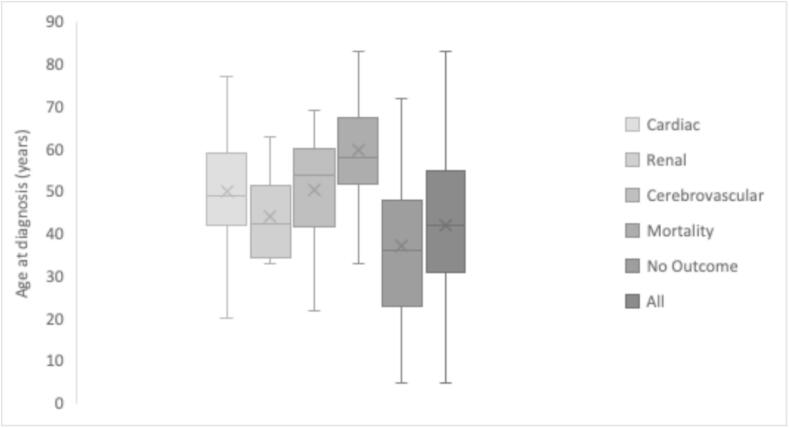
Age at diagnosis across study groups: Box and whisker chart comparing baseline age at diagnosis for groups: cardiac, renal, cerebrovascular, mortality, no outcome and all.

The median age at diagnosis varied across groups, with patients in the CV outcome group 50 (42–59), renal outcome 44.0 (35.5–51), cerebrovascular outcome 53 (41–59), mortality 57.0 (51–66), and those with no outcome 36.0 (23–47). Patients with recorded CV, renal, and cerebrovascular outcomes and mortality had a higher age at diagnosis compared to those with no outcomes (see Fig. 2) (*p* < 0.00001).

### 3.4 MSSI at baseline

**Fig. 3:**
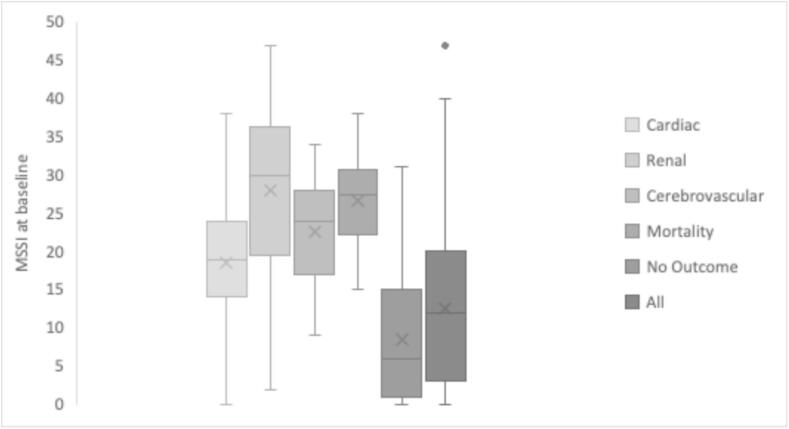
Baseline median MSSI scores across groups: Box and whisker chart comparing baseline MSSI scores for groups: cardiac, renal, cerebrovascular, mortality, no outcome and all.

Baseline MSSI was evaluated across different patient groups, revealing significant differences in severity levels. Patients who experienced cardiac, renal, cerebrovascular outcomes and mortality had a higher median MSSI at diagnosis compared to those with no outcomes (Fig. 3) (*P* < 0.00001).

### 3.5 Molecular analysis

**Fig. 4:**
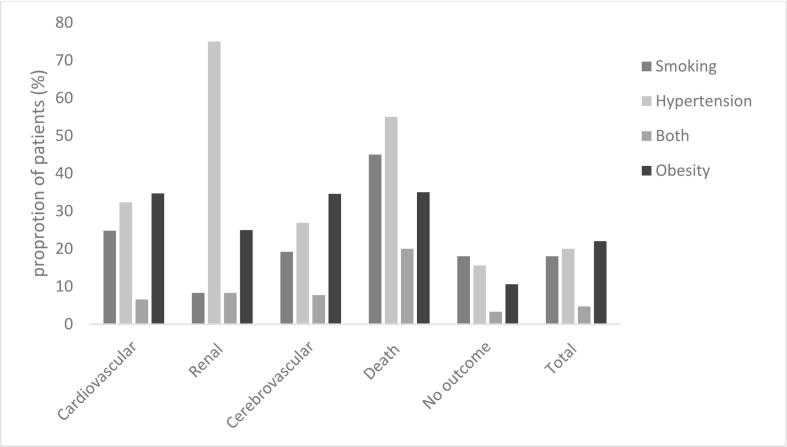
Proportion of patients by outcome and risk factors: The X-axis represents the outcomes, while the Y-axis shows the number of patients as a percentage for each outcome and risk factor.

Of the cohort, 32.8 % (133) had the c.644 A > G (p.N215S) mutation, with a median age at diagnosis of 35.5 years (range, 46–58 years). The c.902G > A (p.R301Q) mutation was present in 6.7 % (27) of patients, with a median age at diagnosis of 53 years (43–64). The median MSSI at baseline was comparable across both mutations, with a slight increase for those with c.902G > A (p.R301Q). Others consisted of single mutations which occurred between one to five times (see *fig. 4, supplementary material).* A summary of key clinical outcomes stratified by the two most common pathogenic variants; c.644 A > G (p.N215S) and c.902G > A (p.R301Q) has also been included (see *table 4, supplementary material).*

### 3.6 Cardiometabolic risk factors

The frequency of risk factors; smoking, hypertension, both risk factors, and obesity, across different event categories is shown in Fig. 4*.* The use of pharmacotherapies for obesity alone (e.g. GLP-1 agonists) was low in the obesity group, with only 5.6 % (5) having an active prescription. Hypercholesterolaemia was observed in 8.4 % of patients, with a slightly higher prevalence in males (9.3 %) compared to females (7.7 %) (Table 1).

### 3.7 Cardiovascular outcomes

A total of 121 patients had 265 recorded outcomes. CV outcomes occurred in 43.4 % (79) of males and 18.8 % (42) of females. Of the cohort, 72 % (87) were on ERT, and 16.5 % (20) were on chaperone therapy, with 14 patients switching from chaperone to ERT. The median MMSI for the cohort was 19 (14–24). The most common genetic variant observed was c.644 A > G (p.N215S) 38.8 % (47), with c.902G > A (p.R301Q) 9.9 % (12) and c.334C > T (p.R112C) 4.0 % (5). Among patients who experienced an NFCVE, 32.3 % (39) had hypertension, 24.8 % (30) were current or recent ex-smokers, and 6.6 % (8) had both risk factors. The median BMI of the cohort was 26.9 kg/m^2^ (23.8–31.2). 34.7 % (42) had BMI >30.0 Kg/m^2^.

### 3.8 Arrhythmia

#### 3.8.1 Patients with atrial fibrillation (AF)

**Fig. 5:**
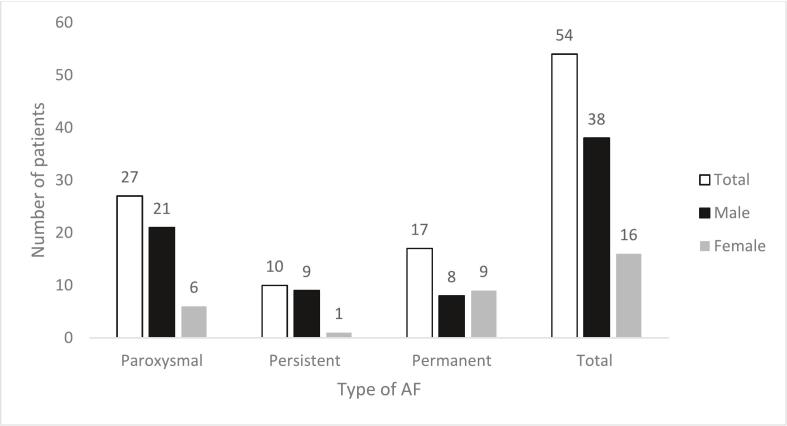
Frequency of AF subtypes by gender: The X-axis represents the different subtypes, while the Y-axis indicates the number of patients. Data is split by male and female patients.

A total of 54 patients experiencing an NFCVE had AF, which was further subdivided into specific AF types: 50 % (27) had paroxysmal AF (PAF), 18.5 % (10) had persistent AF, and 31.5 % (17) had permanent AF. The median MSSI for patients with AF was 18 (14–25), with a median age at diagnosis of 49 years (42–58). The most common genetic mutation among patients with AF was c.644 A > G (p.N215S) 16.7 % (9), followed by c.902G > A (p.R301) 5.6 % (3). (see Fig. 5).

#### 3.8.2 Patients with ventricular tachycardia (VT)

A total of 78 patients who experienced an NFCVE had VT, with 96.2 % (75) having NS-VT and 11.5 % (9) having S-VT, 6 of whom had a prior history of NS-VT. Of the 51 devices placed, 39.2 % (20) were ICD. The median age at diagnosis for these patients was 53 years (43–59), and the median MSSI at baseline was 18 (13–24). Regarding genetic mutations, 39.7 % (31) had the c.644 A > G (p.N215S) mutation, and 9 % (7) had the c.902G > A (p.R301Q) mutation. Most patients 96.2 % (75) had no left ventricular hypertrophy (LVH) at baseline, with 2 having mild and 1 having severe LVH.

### 3.9 Cardiovascular mortality

Among the 15 patients, the median age at diagnosis was 54 years (range, 45–61 years), and the median baseline MSSI was 23.0 (range, 19.5–29.5). Most patients were male, 86.7 % (13), with only 13.3 % (2/15) being female. Regarding cardiometabolic risk factors, 46.7 % (7) had a history of hypertension, 46.7 % (7) were current or recently ex-smokers, while 26.7 % (4) exhibited both risk factors. The median BMI was 27 kg/m^2^, with 40 % (6) classified as obese. Patients who experienced fatal CV events were also included in the total count of CV outcomes (121) if they had prior NFCVE recorded.

20 % (3) carried the c.644 A > G (p. N215S) mutation, and 13.3 % (2) carried the c.902G > A (p. R301Q) mutation. The primary causes of death were heart failure, which accounted for 60 % (9) of cases, cardiac arrest in 20 % (3), MI in 13.3 % (2) and ruptured abdominal aortic aneurysm (AAA) in 6.7 % (1). In the patients who died from heart failure all demonstrated left ventricular systolic dysfunction on echocardiography. Of these, 27 % (4) were receiving a mineralocorticoid receptor antagonist (spironolactone), while none were treated with SGLT2 inhibitors or GLP-1 agonists.

### 3.10 Renal outcomes

Renal outcomes in this cohort have been previously described in detail, including the progression and predictors of FD nephropathy [11]. That analysis identified over 60 % of patients were at lifetime risk of CKD and 3 % progressed to ESKD, with predictors including older age, CV history, higher uACR, and renin–angiotensin–aldosterone system (RAAS) inhibitor use. To avoid redundancy, the present study focused on defined severe renal outcomes. In the cohort of 61.5 % (249/405) with a label of CKD, 8.6 % (35/405) met criteria for stage 3 or worse (eGFR <59 ml/min/1.73m^2^ [32]) at baseline, with a higher prevalence observed in males. Furthermore, a total of 12 patients had 16 defined renal outcomes. Defined renal outcomes were observed in 83.3 % (10) of male patients and 16.7 % (2) of female patients. Of the cohort, 75 % (9) were receiving ERT, while none were on chaperone therapy. The median MSSI for the cohort was 30 (20–35.5). Genetic analysis revealed eleven distinct mutations, including one case each of the c.644 A > G (p.N215S) and c.902G > A (p.R301Q) mutations.

Among patients who had recorded renal outcomes, 75 % (9) had a history of hypertension, 8.3 % (1) were current or recent ex-smokers, and 8.3 % (1) had both risk factors. The median BMI of the cohort was 25.9 kg/m^2^ (22–28.8). 25.0 % (3) had a BMI >30 kg/m^2^. None were treated with SGLT2 inhibitors or GLP-1 agonists.

### 3.11 Cerebrovascular outcomes

A total of 26 patients had 26 recorded cerebrovascular outcomes. Cerebrovascular outcomes were observed in 8.8 % of males (16/182) and 4.5 % of females (10/223), indicating that males in the cohort were approximately twice as likely to experience cerebrovascular events. Regarding therapy, 69.2 % (18) of the patients were on ERT, 19.2 % (5) were on chaperone therapy, and 2 patients had transitioned from chaperone therapy to ERT. Genetic analysis revealed two cases of the c.644 A > G (p.N215S) mutation and two cases of the c.902G > A (p.R301Q) mutation. In terms of risk factors, 26.9 % (7) had hypertension, 19.2 % (5) of were current or recently ex-smokers and 7.7 % (2) had both risk factors. Median BMI of the cohort was 25.9 kg/m^2^ (22.4–31.1). 34.6 % (9) had a BMI >30 kg/m^2^.

Of the 26 cerebrovascular events, 73.1 % (19) were CVAs, and 26.9 % (7) were TIAs. None of the patients who had a CVA had a prior TIA.

### 3.12 Mortality

Among 35 patients, 57.1 % (20) experienced a non-CV cause of death, while 42.9 % (15) had CV mortality. Of the patients who died from non-CV causes, 70 % (14/20) were male, and 30 % (6) were female. In terms of therapy, 90 % (18) were on ERT, and 5 % (1) were on chaperone therapy. The median age at death for males (27) was 61 (range, 56–65), and females (8) was 72 (range, 69–78). Among patients still alive and under follow-up, the median age at diagnosis was 40 (29.5–54), and their median age at last review was 49 (38–61), with an overall age range at last follow-up of 18–84 years.

55 % (11) had hypertension, 45 % (9) were current or recently ex-smokers, and 20 % (4) had both risk factors. The median BMI of this group was 25.2 kg/m^2^ (22.6–33.6), with 35 % (7) classified as obese. The recorded causes of death on the certificate were varied, with the most common being sepsis, 25 % (5), followed by cancer, 20 % (4). Patients who died exhibited a significant burden of cardiac, renal, and cerebrovascular events, contributing to their overall morbidity (see *table 5 and 6, supplementary material*). Among the five patients with sepsis listed as the primary cause of death, three cases were due to community-acquired pneumonia, one to ascending cholangitis, and one to a urinary tract infection. There was no documentation of urinary catheter use in the patient with a UTI, and no evidence suggesting catheter-associated sepsis in any case.

Molecular analysis revealed that the most common variant among FNCVE patients was c.644 A > G (p.N215S), present in 30 % (6) of cases. The second most common variant was c.902G > A (p.R301Q), detected in 20 % (4) of cases.

## 4 Discussion

This analysis highlights the significant prevalence, morbidity, and mortality associated with the c.644 A > G (p.N215S) variant, particularly in males, and its frequent occurrence among those with severe CV outcomes. The typically late onset of symptoms, often accompanied by minimal non-cardiac involvement at baseline, underscores the importance of heightened clinical awareness and early screening. Even patients presenting with mild symptoms may be at risk of serious complications. Cardiac manifestations have been reported as early as the first decade of life [33], reinforcing the need for genetic screening to identify individuals with the p.N215S mutation and to facilitate timely diagnosis and intervention. Cascade screening remains a key strategy in improving outcomes for affected individuals. Notably, over one-third of patients without a recorded clinical outcome harboured the c.644 A > G (p.N215S) variant. These individuals were predominantly female, diagnosed at a younger age, and had lower baseline MSSI scores. Our findings are broadly consistent with those reported in the Fabry Outcome Survey (FOS), particularly with respect to CV morbidity and male predominance in mortality [27,28]. However, our cohort had a higher proportion of patients with the p.N215S mutation and a notable burden of arrhythmia, which may be attributed to referral patterns favouring CV presentations and enhanced rhythm surveillance at our centre. As previously described, the p.N215S variant is associated with a later-onset, cardiac-predominant phenotype and residual enzyme activity, resulting in slower disease progression compared to classical mutations. Enrichment of such patients in our cohort may have been further influenced by cascade screening, which likely identified less symptomatic individuals. These factors introduce selection bias and may explain the predominance of CV over severe renal manifestations in this cohort. Additionally, patients without defined outcomes were significantly younger at diagnosis and female, highlighting age and sex as critical factors in disease progression and outcome risk. The observation that some patients with defined outcomes were not receiving treatment reflects historical variations in treatment eligibility, diagnostic practices, and patient-specific considerations, such as asymptomatic disease or contraindications. Similarly, untreated patients without outcomes often had minimal disease burden and did not meet treatment criteria during the study period.

The relatively high age at diagnosis, even among those with severe outcomes, likely reflects delayed recognition of later-onset phenotypes such as p.N215S, coupled with the non-specific nature of early symptoms and historical limitations in awareness and access to genetic testing. Inclusion of younger, pre-symptomatic individuals (particularly through cascade screening) may have contributed to lower event rates and introduced heterogeneity in disease severity across the cohort. Changes in diagnostic and referral practices over the two-decade study period likely influenced this variability. Our use of age at confirmed diagnosis, rather than age at first symptom or outcome, may underestimate diagnostic delay. While outcome age data were available in some cases, inconsistency in documentation precluded reliable stratification by sex or baseline MSSI, underscoring the need for prospective data collection to better capture diagnostic timelines and disease trajectories. We found that greater baseline disease severity, reflected by higher MSSI scores, was associated with increased risk of CV, severe renal, and cerebrovascular outcomes, as well as mortality. The highest baseline MSSI scores were observed in patients with adverse severe renal outcomes. These findings support the utility of the MSSI as a predictive tool for future morbidity, warranting further validation in prospective studies. Although cerebrovascular events were reliably documented and contributed to the neurological domain of the MSSI, incomplete recording of neuropathic pain and peripheral symptoms (particularly in earlier records) may have led to underestimation of scores in this domain. To avoid potential bias, domain-specific MSSI analyses were not conducted.

Renal outcomes in this cohort have been previously described in detail, including progression patterns and predictors of Fabry nephropathy [11]. That analysis identified a high lifetime risk of CKD, with a subset of patients progressing to ESKD. Key predictors of renal decline included older age, CV history, elevated urine albumin-to-creatinine ratio (uACR), and use of RAAS inhibitors. To avoid redundancy, the present study focused on defined severe renal outcomes. Although a substantial proportion of patients carried a clinical diagnosis of CKD, only a minority met criteria for moderate disease at baseline (based on eGFR), with a clear male predominance. Furthermore, only a small number experienced severe renal events such as dialysis or transplant, supporting the observation that such complications were less frequent than CV outcomes in this cohort (for reasons discussed).

Cardiometabolic risk factors were prevalent in this cohort and appeared to influence clinical outcomes. Hypertension, smoking, and obesity were common among patients with adverse events and likely contributed to increased risk of heart failure, MI, and other CV complications. Ongoing smoking status could not be fully assessed due to limited longitudinal data, a limitation of this retrospective analysis. Importantly, none of the patients who died from heart failure were receiving SGLT2 inhibitors or GLP-1 receptor agonists, indicating a potential gap in therapeutic optimisation. Although evidence supporting their use in FD is limited [34], their established efficacy in managing heart failure in other populations [35,36] supports further investigation. The low uptake of pharmacotherapies for obesity likely reflects their recent introduction and limited indications during much of the study period. At the time, use of SGLT2 inhibitors and GLP-1 receptor agonists in the UK was generally restricted to patients with type 2 diabetes, heart failure with reduced ejection fraction, or obesity, which may have constrained access in this cohort. Despite proposed mechanistic benefits [37], further studies are warranted to evaluate their role in FD. Diabetes was recorded only if patients were prescribed antidiabetic medications. Consequently, individuals with undiagnosed or untreated diabetes may not have been captured. The low prevalence observed may reflect under recognition or incomplete documentation rather than true absence of disease. Future studies incorporating HbA1c and fasting glucose will be important for assessing the metabolic burden further. Given the complexity of multi-morbidity, comprehensive care, including early diagnosis, risk factor modification, and targeted pharmacotherapy remains essential to improving outcomes [27,38]. Hypercholesterolaemia is another recognised contributor to CV risk in FD [39,40]. In our cohort, hypercholesterolaemia was present in 8.4 % of patients. However, the lack of consistent serial lipid data precluded analysis of treatment effects or temporal trends. Total cholesterol is a crude marker of lipid status; lipoprotein subfractions such as low density lipoprotein (LDL), high density lipoprotein (HDL), and triglycerides may be variably affected by Fabry-related pathology and renal dysfunction. Previous work has demonstrated that lipid abnormalities may persist despite ERT [38], further supporting the need for lipid-focused therapeutic strategies in this population.

Patients who died in our cohort exhibited a higher burden of multi-morbidity and cardiometabolic risk factors. Mortality analysis showed that non-CV causes accounted for 54 % of male and 71 % of female deaths, often in the context of pre-existing cardiac, renal, and cerebrovascular disease. The leading causes of death were sepsis and cancer, underscoring the breadth of health risks faced by patients. These findings highlight the importance of multidisciplinary management and attention to comorbidities. The observed sex difference in life expectancy, with males having significantly shorter survival, aligns with prior reports from the Fabry Registry [6]. This calls for earlier recognition and diagnosis of FD, particularly in individuals with non-specific symptoms. The significant CV and renal involvement present at diagnosis in many patients suggests missed opportunities for early intervention. Enhancing clinician awareness, promoting cascade screening, and exploring newborn screening strategies may facilitate earlier identification and timely initiation of treatment.

We also found a high prevalence of arrhythmias, including NS-VT and AF. To our knowledge, this is the first study to characterise AF subtypes in adults with FD. The frequency of both paroxysmal and persistent AF suggests that standard 24-h ECG monitoring may be insufficient, and extended or event-triggered monitoring should be considered. The strong association between arrhythmia burden and the c.644 A > G (p.N215S) variant, which is linked to severe CV outcomes, warrants further research into targeted monitoring and management strategies [41,42,43].

### 4.1 Strengths and limitations

This retrospective cohort study benefits from a large sample size and extended follow-up, enabling robust evaluation of real-world clinical outcomes over more than two decades. It offers a comprehensive assessment of disease burden and progression in FD, encompassing predominantly CV, severe renal, and cerebrovascular events, as well as CV and all-cause mortality. The semi-longitudinal nature of data collection, with multiple events recorded per patient over time, provides valuable insights into disease trajectory. However, the retrospective design introduces potential information bias due to reliance on the completeness and accuracy of historical medical records. While the study employed a cohort approach, variability in follow-up duration and documentation limited the ability to apply a standardised longitudinal framework, and outcomes were analysed using primarily cross-sectional methods. A further limitation of the study design is susceptibility to confounding and the absence of a control group, which limits the ability to infer causal relationships between variables and outcomes. Selection bias may be present due to referral patterns favouring patients with cardiac phenotypes, and the inclusion of individuals identified through cascade screening may have enriched the cohort with later-onset or milder cases. The precise contribution of cascade testing could not be quantified due to incomplete family linkage documentation.

## 5 Conclusion

This study provides real-world observational insights into the clinical trajectories of patients with FD over two decades. A substantial proportion of patients experienced defined CV, renal, or cerebrovascular outcomes, with older age at diagnosis and higher baseline MSSI more frequently observed among those with adverse events. The high prevalence of the c.644A > G (p.N215S) variant (particularly among males with CV complications) illustrates the importance of genetic characterisation in clinical evaluation. Cardiometabolic risk factors such as hypertension, smoking, and obesity were commonly present in those with poorer outcomes, highlighting the potential value of systematic risk factor assessment and management. The frequent detection of arrhythmias, especially paroxysmal AF, supports the need for enhanced cardiac monitoring protocols. The low uptake of cardiometabolic therapies in at-risk patients may reflect an underutilised opportunity for intervention. Overall, these findings support a comprehensive, individualised approach to care, including early diagnosis, CV risk assessment, and targeted multidisciplinary management to optimise outcomes.

## CRediT authorship contribution statement

**Eamon P. McCarron:** Writing – review & editing, Writing – original draft, Data curation, Conceptualization. **Rajkumar Chinnadurai:** Writing – review & editing, Writing – original draft, Formal analysis, Data curation. **Jonathan Meyer:** Writing – review & editing. **Thomas Anderson:** Data curation. **Karolina M. Stepien:** Writing – review & editing. **Reena Sharma:** Writing – review & editing. **Peter Woolfson:** Writing – review & editing. **Ana Jovanovic:** Supervision, Methodology, Funding acquisition, Data curation, Conceptualization.

## Declaration of competing interest

The authors declare no conflict of interest.

## Data Availability

Data will be made available on request.

## Appendix A Supplementary data

Supplementary material Image 1
