# Supplementary material for: Real-world clinical outcomes in adult patients with Fabry disease: A 20-year retrospective observational cohort study from a single centre
Source: Mol Genet Metab Rep. 2025 May 14;43:101229. doi: 10.1016/j.ymgmr.2025.101229 (PMC12144493; doi:10.1016/j.ymgmr.2025.101229)
Supplement: Supplementary file 1 — Supplementary material [file mmc1.docx]

**Supplementary data**

**Figure 4 Frequency of mutations in 405 patients with Fabry disease:** X-axis represents different mutations, while the Y-axis shows the number of patients exhibiting each mutation.

|  | **c.644A>G (p.N215S)**  133 (32.8%) | **c.902G>A (p.R301Q)**  27 (6.7%) | **Other**  245 (60.5%) |
| --- | --- | --- | --- |
| **Age at diagnosis** | 35.5  (46.0-58.0) | 53.0  (43.0-64.0) | 37.0  (24.0-48.0) |
| **MSSI at baseline** | 11.0  (1.5-18.0) | 15.0  (11.0-21.0) | 13.0  (3.0-20.5) |
| **Cardiovascular (CV)** | 47  (35.3%) | 12  (44.4%) | 62  (25.3%) |
| **Renal** | 1  (0.8%) | 1  (3.7%) | 10  (4.1%) |
| **Cerebrovascular** | 2  (1.5%) | 2  (7.4%) | 22  (8.9%) |
| **CV mortality** | 3  (2.3%) | 2  (7.4%) | 10  (4.1%) |
| **Non-CV mortality** | 6  (4.5%) | 4  (14.8%) | 10  (4.1%) |

**Table 4** Distribution of cardiovascular, renal, cerebrovascular outcomes, and mortality by mutation

| **Cause of death** | **N** | **Age at diagnosis** | **Age at death** | **MMSI** |
| --- | --- | --- | --- | --- |
| Cardiovascular | 15 | 54 (45.5-61) | 63.0 (57-70.5) | 23.0 (19.5-29.5) |
| Renal | 1 |  |  |  |
| Cerebrovascular | 0 |  |  |  |
| Other  **Total** | 20  35 | 57.0 (51-66)  56.0 (49.5-63) | 65.0 (58-71)  65.0 (58-7) | 27.5 (22.5-30.5)  25.0 (21-30.5) |

**Table 5** Cause of Death and associated patient information

| Outcome | All-cause mortality (35) | Non-CV mortality (20) |
| --- | --- | --- |
| CV | | |
| N | 20 | 15 |
| Events | 20 | 15 |
| **NFCVE**  **Arrythmia**  **AF**  **NS-VT**  **S-VT**  **MI**  **Heart failure**  **(NYHA III/IV)**  **Cardiac device**  **ICD**  **PPM** | 12  4  6  2  2  1  4  5 | 7  4  1  1  2  1  1  2 |
| Renal | | |
| N | 9 | 7 |
| Events | 9 | 7 |
| **HD/PD**  **RT** | 6  3 | 4  3 |
| Cerebrovascular |  |  |
| N | 5 | 5 |
| Events | 5 | 5 |
| **CVA**  **TIA** | 4  1 | 4  1 |

**Table 6 Patient outcomes by mortality status:** Number of patients who died and outcomes for cardiovascular, renal, and cerebrovascular conditions, comparing all-cause mortality (N=35) with those experiencing non-CV mortality (N=20). *Note: This table summarises the clinical burden of prior cardiovascular, renal, and cerebrovascular events in patients who died during the study period. It does not indicate the cause of death.*
